# Advice after urgent suspected cancer referral when cancer is not found in England: Survey of patients’ preferences and perceived acceptability

**DOI:** 10.1016/j.pmedr.2024.102781

**Published:** 2024-06-10

**Authors:** Ruth E.C. Evans, Harriet Watson, Jo Waller, Brian D. Nicholson, Thomas Round, Carolynn Gildea, Debs Smith, Suzanne E. Scott

**Affiliations:** a Wolfson Institute of Population Health, Queen Mary University of London, UK; b King’s College London, UK; c Guy’s and St Thomas’ NHS Foundation Trust, UK; d Nuffield Department of Primary Care Health Sciences, University of Oxford, UK; e National Disease Registration Service, NHS England, UK

**Keywords:** Cancer, Neoplasm, Early Diagnosis, Early Detection, Cancer Screening, Opportunistic intervention, Patient Survey, Health promotion, Public Health

## Abstract

**Objective:**

No standardised approach exists to provide advice after urgent suspected cancer (USC) referral when cancer is not found. This study aimed to assess preferences and acceptability of receiving advice after USC referral related to: 1) managing ongoing symptoms, 2) responding to early symptoms of other cancers, 3) cancer screening, 4) reducing risks of future cancer.

**Methods:**

2,541 patients from two English NHS Trusts were mailed a survey 1–3 months after having no cancer found following urgent suspected gastrointestinal or head and neck cancer referral. Participants were asked about: willingness to receive advice; prospective acceptability; preferences related to mode, timing and who should provide advice; and previous advice receipt.

**Results:**

406 patients responded (16.0%) with 397 in the final analyses. Few participants had previously received advice, yet most were willing to. Willingness varied by type of advice: fewer were willing to receive advice about early symptoms of other cancers (88.9%) than advice related to ongoing symptoms (94.3%). Acceptability was relatively high for all advice types. Reducing the risk of future cancer advice was more acceptable. Acceptability was lower in those from ethnic minority groups, and with lower levels of education. Most participants preferred to receive advice from a doctor; with results or soon after; either face to face or via the telephone.

**Conclusions:**

There is a potential unmet need for advice after USC referral when no cancer is found. Equitable intervention design should focus on increasing acceptability for people from ethnic minority groups and those with lower levels of education.

## 1 Introduction

Urgent suspected cancer (USC) referral pathways, mostly organised by anatomical site, facilitate patient access to specialist investigation. In England, the National Health Service (NHS) waiting time target is that patients suspected of having cancer should have cancer ruled out or receive a diagnosis within 28 days of USC referral (NHS England, 2019). In England, in 2022–2023, nearly 3 million patients were referred via USC pathways (NHS England, 2023). Most of those referred (93 %) did not have cancer diagnosed at that time (Office for Health Improvements and Disparities, 2022). However, there is evidence that this group of patients may be at risk of future cancer either because of missed cancers or due to common risk factors (e.g. diet or smoking) (Scott et al., 2020, Nielsen et al., 2018). Analyses of national cancer data found 1,338 subsequent cancers per 100,000 USC referrals per year in years 1–5 after cancer had been ruled out in year 1, higher than expected based on age and sex (Scott et al.). There is also evidence that patients may delay seeking help for symptoms after USC referral when no cancer has been found (Renzi et al., 2015, Renzi et al., 2016). This could be due to concerns about re-consulting the doctor because of worries about wasting doctors time, or appearing hypochondriacal. There may also be uncertainty about appropriate next actions (Barnett et al., 2018, Barnett et al., 2017) or false reassurance from the 'all-clear' result leading to subsequent symptoms being interpreted as benign. To date, little focus has been given to this large patient group, and much is unknown about their emotional and behavioural responses following USC referral (Rowlands et al., 2022).

There may be scope to provide patients with additional advice following USC referral when cancer is not found. This could include providing tailored brief information to avoid missing cancers at other anatomical sites as well as to ensure subsequent cancers are diagnosed at an early stage, or even prevented. The latter could be achieved through raising cancer awareness as well as facilitating health behaviour change. A USC referral is a potentially under-utilised ‘teachable moment’ when people are more responsive and receptive to health information (Tang et al., 2014). Previously in an NHS pilot study, a brief smoking cessation intervention was offered to patients following head and neck USC referral. Seventy-eight percent of smokers (n = 80) accepted a referral to stop smoking services at the consultation and of these, when contacted at least four months later, 36 % (n = 29) reported that they had quit smoking, at least temporarily. A reduction in smoking following cancer assessment has been observed in other contexts: in a randomised lung cancer screening pilot study, Brain et al (Brain et al., 2017) reported an increased likelihood of smoking cessation in people who had received screening compared to the non-screened control group; both groups received the same quit smoking support prior to randomisation.

The NHS has already made it a standard of care that health care professionals offer brief advice opportunistically to encourage healthier behaviour (Making Every Contact Count) (National Health Service. NHS Standard Contract, 2018, Health Education England, 2022). This approach could be tailored to the USC referral context when cancer is not found. Box 1 summarises four key primary and secondary cancer prevention strategies that could be appropriate in this context (NHS England, 2022).

Box 1 **Summary of four types of advice that could be given to patients after USC referral when no cancer is found.**

**1) Advice about what to do about on-going symptoms.**

Examples of this include: when to contact a doctor or other HCP if symptoms don’t go away, what to do about new symptoms, how to manage ongoing symptoms and what to do if they get worse, who to contact about ongoing symptoms, how to contact someone and why it is important to get advice.

**2) Advice about spotting early symptoms of different types of cancer.**

Examples of this include: early symptoms, how to spot changes in your body, why it is important to seek help quickly for different signs of cancer, how to seek help for possible signs of cancer, tackling worries or concerns you might have about getting help.

**3) Advice about cancer screening.**

Examples of this include: checking you are up-to-date with cancer screening, help getting a screening kit or screening appointment if needed, why cancer screening is important, advice about bowel, breast or cervical screening.

**4) Advice about how to reduce the chances of developing cancer (reducing risk of future cancer)**

Examples of this include: personal risk (likelihood) of developing cancer in the future, the types of cancer you are most at risk of developing, how to stop tobacco use, make diet changes, increase the amount of exercise, or reduce the amount of alcohol you drink to reduce the chances of developing cancer.

Prior to developing and advocating for this additional advice, it is vital to investigate patients’ perspective of acceptability and willingness (Skivington et al., 2021). The Theoretical Framework of Acceptability (Sekhon et al., 2017) defines acceptability as: “A multi-faceted construct that reflects the extent to which people delivering or receiving a healthcare intervention consider it to be appropriate, based on anticipated or experienced cognitive and emotional responses to the intervention”. Acceptability can be measured concurrently whilst participating in a health intervention, before participation (prospective) or after (retrospective).

Previous studies have measured feasibility, willingness, and acceptability of lifestyle advice or advice about other cancers, following cancer screening (Stevens et al., 2019, Stevens et al., 2019, Scott et al., 2021, Sinclair et al., 2019, Bamidele et al., 2022, Anderson et al., 2021, Anderson et al., 2014, Harvie et al., 2021). These studies have generally concluded that patients find advice in this context acceptable, with cancer screening seen as an opportunity to learn more about cancer or make changes to reduce future cancer risk. Non-white ethnicity, greater cancer risk factor awareness, gender and higher levels of educational attainment were associated with greater willingness to receive lifestyle advice at cancer screening (Stevens et al., 2019, Stevens et al., 2019). People who had not previously engaged with screening were less willing to receive advice (Scott et al., 2021) and others stated they would be less likely to attend screening if offered lifestyle advice (Stevens et al., 2019, Stevens et al., 2019).

At present we do not know patients’ views concerning advice linked to USC referral pathways after cancer has been ruled out. Unlike people who have been through cancer screening, these symptomatic patients may differ in receptivity or have different priorities to screening invitees. As a first step in intervention development, the objective of this study was to measure patients’ current receipt of advice, their willingness to receive advice after USC referral when no cancer is found, and to investigate if acceptability differs between socio-demographic groups.

## 2 Methods

### 2.1 Study design

The study was an observational cross-sectional survey of NHS patients. Details about the study protocol were approved by the South Central − Berkshire B Research Ethics Committee [REC ref: 22/SC/0239] and registered in advance of data collection at www.clinicaltrials.gov [ref: NCT05479851].

### 2.2 Participants

Patients were eligible to participate if they were ≥ 18 years old, had been referred through an USC pathway and had no cancer found within the past 1–3 months. Patients were recruited from 3 USC referral pathways: Upper Gastrointestinal (Upper-GI), Lower Gastrointestinal (Lower-GI), and Head and Neck, from two NHS Trusts in London, England. These pathways have a more even gender balance than some suspected cancer referral pathways and have large numbers of referrals. Patients were excluded if they were diagnosed with cancer following referral, or within 3 months of receiving the invitation to participate in this study, or discharged from the pathway for other reasons (e.g. did not attend appointments).

### 2.3 Procedure

#### 2.3.1 Patient identification and recruitment

Eligible patients were sent an invitation, information sheet and questionnaire by their direct care team between October 2022 and January 2023. The questionnaire was also available online (powered by Qualtrics, Provo UT). Consent was assumed by the return of the questionnaire. One reminder was sent to non-responders, a minimum of three weeks after posting. Participation was anonymous: each eligible patient was assigned an ID code. A code break sheet linking patients’ names, addresses and ID code was destroyed after reminder letters were sent. To enable assessment of participation bias, the NHS trusts provided aggregate data for all invited patients: gender, age, and interval between date of being informed about outcome of USC referral and date of questionnaire posting.

### 2.4 Materials and measures

Four ‘types of advice’ (see Box 1) were presented to participants. The questionnaire (developed specifically for use in this study, see Supplementary file) measured responses to these types of advice. The measures were as follows.

#### 2.4.1 Willingness to receive advice

Willingness to receive advice was measured using a question adapted from Stevens et al (Stevens et al., 2019) previously used in general population surveys e.g: *“Would you have been willing to receive advice about [on-going symptoms], sometime after your urgent referral appointment?”* with response options from 1 “No, definitely not” to 5 “Yes, definitely”. For analyses, the 5 categories were collapsed into binary categories “willing” (4–5) and “not-willing” (1–3), for ease of comparison with other studies (Stevens et al., 2019). Participants were also asked to indicate if they would want to receive specific examples of each of the types of advice (response options “yes”, “no”, “n/a”) and to report if they had received this advice (response options “yes”, “no”, “unsure”).

#### 2.4.2 Prospective acceptability

Prospective acceptability was measured for each type of advice using thirteen items with five-point Likert scale responses (1 “Strongly disagree” to 5 “Strongly agree”) designed to capture the domains specified in the Theoretical Framework of Acceptability (TFA) (Sekhon et al., 2017). Items were grouped into two sub-scales based on psychometric testing in a previous study of women with experience of breast screening: cognitive and affective acceptability (Scott et al., 2021). *Cognitive acceptability* included eight items reflecting the TFA domains of: perceived burden, coherence, opportunity costs and efficacy of the intervention. Scores could range from 8 to 40 with higher scores indicating higher cognitive acceptability. *Affective acceptability* (how an individual feels about an intervention) includes five items, with higher scores (range: 5–25) indicating higher affective acceptability. There was good subscale internal reliability for the scales with Cronbach's alpha ranging from 0.833 to 0.891.

#### 2.4.3 Preferred delivery of advice

For each type of advice, participants were asked to indicate their preferences about how it was delivered, by answering “yes” or “no” to different options related to mode (e.g. “text message with links to videos and or websites”; “phone appointment with a health care professional”), timing (e.g. “with my urgent referral results”; “2–4 weeks after receiving my results”) and health care professional (e.g. “a doctor at the USC referral clinic”).

#### 2.4.4 Socio-demographic variables, health and health-related behaviours

Participants were asked to confirm their marital status, ethnic group and highest level of education. They also provided their age and postcode. Postcode was converted into index of multiple deprivation decile (Ministry of Housing, Communities Local Government Index of Multiple Deprivation, 2019) and collapsed into binary categories for analyses (deciles 1–5 higher deprivation; 6–10 lower deprivation).

Participants were asked to indicate comorbidities (including cancer and time since diagnosis), and to provide an estimate of the number of GP appointments in the last year. They were also asked about their health-related behaviours including bowel cancer screening in the last two years, fruit and vegetable consumption, smoking status, alcohol consumption (using the AUDIT-C Scale (Bush et al., 1998, UK Government Alcohol use disorders identification test consumption, 2023) and physical activity.

### 2.5 Statistical analysis

Paper questionnaires were manually entered into SPSS for analyses (version 28) and 10% were double entered to check data entry quality (over 90% concordance). Online questionnaires were uploaded into SPSS from Qualtrics. Imputation for missing data using median values at an individual item level was completed for the acceptability scales when a participant had completed at least 50% of each sub-scale. The proportion of the sample for each sub-scale with imputed values varied from 2% to 9%. Imputation had minimal impact on mean/median values. Throughout, proportions were calculated with the dominator being all participants who answered the questions excluding those who said “n/a”.

T-tests and Chi squared tests were used to assess whether patients who took part differed from those who did not in terms of: age, gender and time interval between being informed of the outcome from USC referral and posting date of the study invitation letter.

Chi squared tests (or Fisher’s Exact test where cell numbers did not meet the minimum required) were used to examine differences by USC pathway in willingness to receive advice. Non-parametric tests of variance were used to compare differences in willingness to receive advice between the different advice types (Cochran Q test between all four types, McNemar test between pairs), and differences in prospective acceptability ratings (Friedman test across all four types, Wilcoxon test between pairs). Socio-demographic factors, USC pathway, health and health behaviour factors were entered into univariate linear regression to assess associations with prospective acceptability. Factors significantly associated with acceptability (p < 0.05), for at least once advice type, were then included in multivariable linear regression analyses which included data assumption checks to ensure analysis validity.

Sample size calculations indicated n = 115 participants per pathway were required to find a medium difference (14%) in willingness between USC referral pathways, with 80% power at 5% level of significance if approximately 89% of the sample would be willing to receive advice. This power calculation was based on interim analyses (as there was no comparable studies in this population) and is sufficient for all other planned analyses. Where appropriate, p-values were adjusted using Bonferroni Correction to reduce the risk of type 1 error (Sedgwick, 2014).

## 3 Results

2,541 patients were mailed a study invitation. 406 (16%) responded to the invitation, 365 completed a paper questionnaire and 41 used the online version. 397 were included in the final analyses (9 were excluded due to a cancer diagnosis within 3 months of receiving the questionnaire). Table 1 summarises the characteristics of participants. Patients who chose to participate in the study were older than those who did not: mean age 62.4 years (sd: 16.0) compared to 58.8 years (17.4) [t(6 0 1) = 4.10, p < 0.001]. There was no significant difference in gender [*X*^2^(1, 2541) = 0.343, p = 0.558] or the time interval between confirmation of “no cancer found” and the date of posting of the invitation [t(5 3 0) = 0.016, p = 0.986] between those who returned a completed questionnaire and those who did not (see Supplementary Table S1).

**Table 1:** Characteristics (count, percentage) of patients who had been referred on an urgent suspected cancer pathway with no cancer found who responded to a survey between October 2022 and January 2023 (n = 397 unless otherwise noted).

**Characteristic**		**n**	**%**
**Site**	1	281	71.0
	2	116	29.0
**USC Pathway**	Lower GI	247	62.2
	Upper GI	57	14.4
	Head & Neck	93	23.4
**Mean Age**		62.37 years (sd: 16.04)	
**Gender**	Male	177	43.8
	Female	228	56.2
**Ethnic Group** (n = 386)	White	289	74.9
	Other ethnic groups	97	25.1
	Asian/ Asian British	41	10.6
	Black, Black British, Caribbean, African	34	8.8
	Mixed or multiple ethnicities	16	4.1
	Other	6	1.6
**Education** (n = 363)	No qualifications/ GCSE/ O Level/ CSE/ Vocational qualifications (NVQ1 + 2)	158	43.5
	A Level or equivalent (NVQ 3)	47	13.0
	Bachelor degree or equivalent (NVQ 4)/ Masters/ PhD or equivalent	158	43.5
**Marital Status** (n = 370)	Single	102	27.6
	Married or civil partner	203	54.9
	Separated or divorced	32	8.6
	Widowed	33	8.9
**IMD** (n = 295)	Higher deprivation (deciles 1–5)	181	61.4
	Lower deprivation (deciles 6–10)	114	38.6
	1 (Most deprived quintile)	33	11.2
	2	112	38.0
	3	60	20.3
	4	51	17.3
	5 (least deprived quintile)	39	13.2
**Previous cancer diagnosis** (n = 378)	Yes	68	17.1
**Co-morbidity**	At least one co-morbidity	305	76.8
	Heart disease	56	14.3
	Lung disease or breathing condition	64	16.5
	Joint or bone condition	141	35.5
	Diabetes	72	18.0
	Gastrointestinal condition	159	40.1
	Anxiety or depression	115	29.1
**Screened for bowel cancer in last two years** (n = 370)	Yes	297	80.3
**Mean number of GP visits in the last year** (n = 328)		4.5 visits (sd:4.5)	
**Fruit and vegetable consumption in the last month:** (n = 382)	Less than 5 portions a day	286	74.9
	Five or more portions a day	96	25.1
**Use of tobacco** (n = 385)	Never smoked or used tobacco	192	49.9
	Used to smoke or use tobacco/ Currently smoke or use tobacco	196	50.9
**Mean number of days doing 30 min of physical activity in past week** (n = 382)		3.1 days (sd 2.3)	
**Alcohol use (Audit-C)** (n = 383)	Proportion with a score of 5+	86	22.5

### 3.1 Current receipt of advice versus what advice patients would want, and be willing to receive, after USC referral

**Fig. 1:**
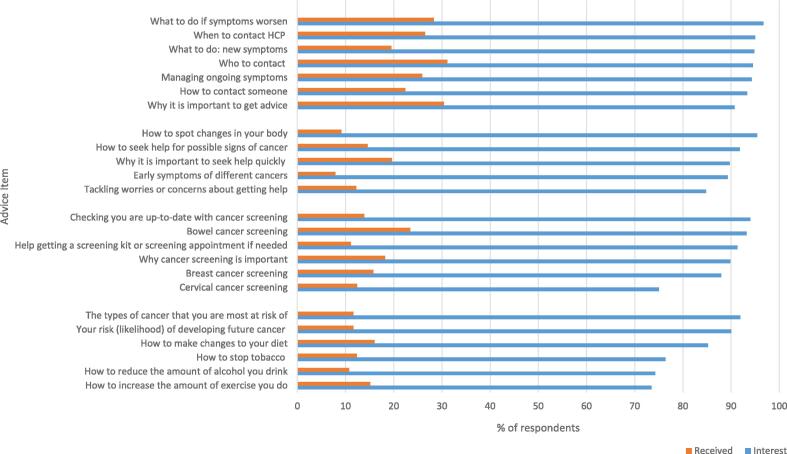
Bar chart of proportion (percentage) of patients who responded to a survey between October 2022 and January 2023 who received vs. wanted to receive different types of advice after urgent suspected cancer referral when no cancer was found.

Far more people wanted to receive advice than had done so since their referral (See Fig. 1). The largest gaps between what advice participants wanted and actual receipt were for the advice items: ‘how to spot changes in your body’ (95.4% versus 9.1% and ‘early symptoms of different types of cancers’ (89.3% versus 7.9%).

The majority (over 88%) of participants were willing to receive advice after a USC referral (see Table 2) but this varied by type of advice (Q = 14.638, p = 0.002). Post-hoc tests indicated fewer participants were willing to receive advice about spotting early symptoms of different cancers compared to advice about ongoing symptoms (88.9% compared to 94.3%, McNemar *X*^2^ = 12.410, p < 0.001). There was no difference in willingness to receive advice between the three USC pathways (see Supplementary Table S2).

**Table 2:** Proportion (count, percentage, confidence intervals) of patients willing to receive advice after urgent suspected cancer referral when no cancer was found who responded to a survey between October 2022 and January 2023.

**Willingness to receive advice about [Type of advice]**				**Dichotomised** **outcome**		
	n	%	95 % Confidence Intervals		%	95 % Confidence Intervals
**Ongoing symptoms** (n = 388)						
No, definitely not	1	0.3	0.0––1.0	Not willing	5.7	3.4–8.1
No, probably not	8	2.1	0.8–3.6			
Not sure	13	3.4	1.6–5.2			
Yes, probably	105	27.1	22.8–31.4	Willing	94.3	91.9–96.6
Yes, definitely	261	67.3	62.9 – 71.9			
**Spotting early symptoms of different types of cancer** (n = 389)						
No, definitely not	6	1.5	0.5–2.9	Not willing	11.1	8.0–14.2
No, probably not	15	3.9	2.1–5.9			
Not sure	22	5.7	3.4–8.2			
Yes, probably	93	23.9	19.6–28.1	Willing	88.9	85.8 – 92.0
Yes, definitely	253	65.0	60.1–69.8			
**Cancer screening** (n = 379)						
No, definitely not	2	0.5	0.0–1.3	Not willing	8.2	5.8–11.0
No, probably not	9	2.4	1.0–4.2			
Not sure	20	5.3	3.2–7.7			
Yes, probably	100	26.4	21.8 – 31.3	Willing	91.8	89.0–94.2
Yes, definitely	248	65.4	60.0–70.3			
**Reducing risk of future cancer** (n = 376)						
No, definitely not	1	0.3	0.0–0.8	Not willing	7.4	4.8–10.3
No, probably not	11	2.9	1.3–4.6			
Not sure	16	4.3	2.4–6.4			
Yes, probably	103	27.4	23.1–31.8	Willing	92.6	89.7–95.2
Yes, definitely	245	65.2	60.5 – 69.7			

The highest proportions of participants wanting to receive advice were for: ‘what to do if symptoms get worse’ (96.7%, n = 354); ‘how to spot changes in your body’ (95.4%, n = 353); and ‘when to contact a doctor or health care professional if symptoms don’t go away’ (95.0%, n = 361). The least interest was expressed for: ‘cervical cancer screening’ (82.0%, n = 188); ‘how to reduce the amount of alcohol you drink, to reduce the chances of developing cancer’ (74.3%, n = 153); and ‘how to increase the amount of exercise you do, to reduce the chances of developing cancer’ (73.5%, n = 264).

### 3.2 Prospective acceptability of receiving advice after an USC referral

Cognitive and affective acceptability were relatively high for all four types of advice (see Supplementary Table S3). Friedman tests indicated that there were significant differences in acceptability between the four types of advice (cognitive acceptability *X*^2^(3) = 9.730, p = 0.021; affective acceptability X^2^(3) = 48.65, p < 0.001). Post-hoc comparisons (with a Bonferroni correction) demonstrated higher affective acceptability for advice about reducing risk of future cancer compared to advice about spotting early symptoms of different cancers (Z = -5.964, p < 0.001), and advice about ongoing symptoms (Z = -3.541,p < 0.001). Advice about cancer screening also had higher affective acceptability compared to advice about spotting early symptoms of different types of cancers (Z = -4.738, p < 0.001). There were no significant post-hoc differences between types of advice for cognitive acceptability after Bonferroni corrections. A summary of individual acceptability items scores for each advice type can be found in Supplementary Table S4.

### 3.3 Factors associated with prospective acceptability of receiving advice after USC referral

Based on the univariate analyses (see Supplementary Tables S5, S6), nine variables (age, USC pathway, ethnicity, marital status, education, fruit and vegetable consumption, exercise frequency, smoking status and alcohol use) were entered into multivariable linear regressions for cognitive and affective acceptability, for each type of advice (see Supplementary Tables S7, S8). After Bonferroni correction, education attainment (degree level or above) was significantly associated with higher cognitive acceptability scores for advice about on-going symptoms (B = 2.568 (sd: 0.674), p < 0.001) and both greater cognitive and affective acceptability of advice about how to reduce the risk of future cancer (cognitive B = 2.476 (0.720), p < 0.001; affective B = 1.690 (0.537), p = 0.002). White ethnicity was significantly associated with higher affective acceptability scores for all types of advice except advice about reducing the risk of future cancer (Ongoing symptoms B = -1.666 (0.582), p = 0.005; Early and common signs B = -1.689 (0.593), p = 0.005, Cancer Screening B = -2.125 (0.571), p < 0.001).

### 3.4 Preferences for delivery of advice after USC referral

Across all four types of advice, most participants indicated that they would like to receive advice from the doctor at the urgent referral clinic (85.4% −91.2%, across the four types of advice) or their GP practice (82.0% −87.3%). Fewer participants indicated that they would like to receive advice from a nurse or other health care professional at their GP practice, especially regarding ongoing symptoms (58.9% −71.8%) (See Supplementary Figure S9).

**Fig. 2:**
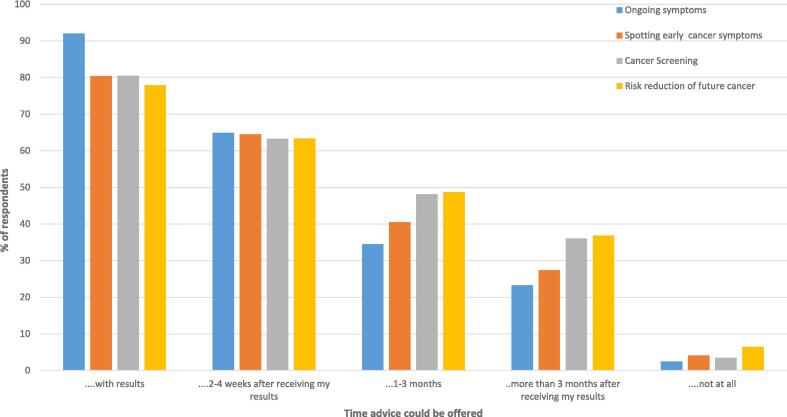
Bar chart of proportion (percentage) of patients who responded to a survey between October 2022 and January 2023 regarding preferences for timing of advice after urgent suspected cancer referral.

**Fig. 3:**
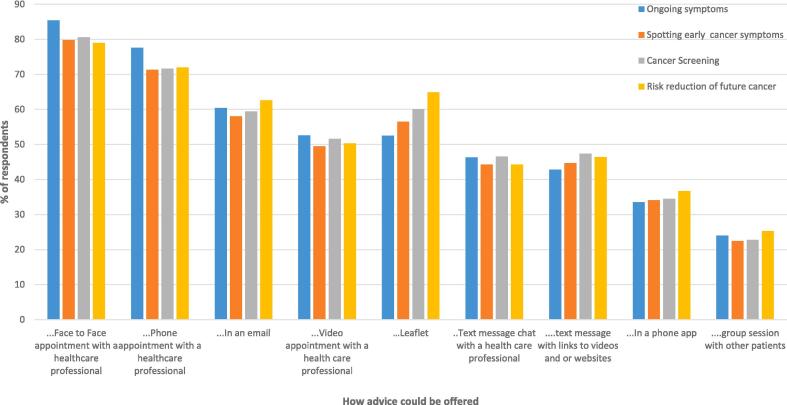
Bar chart of proportion (percentage) of patients who responded to a survey between October 2022 and January 2023 regarding preferences for how to receive advice after urgent suspected cancer referral.

The majority of participants indicated they would like to be given advice at the point of receiving USC referral results (77.9% − 92.0% across the four types of advice), or within the first few weeks (63.2% − 64.5%) (See Fig. 2). Participants would like to be given advice at a face-to-face appointment (79.0% − 85.4%, across the four types of advice), via a telephone appointment (71.3% − 77.6%) or via an email (58.1% − 62.6%). Other methods of digital delivery (e.g. via a phone app) or in a group session were much less popular in comparison (33.5%- 36.7%, and 22.5% −25.3% respectfully) (See Fig. 3).

## 4 Discussion

People with recent experience of USC referral expressed high levels of willingness and prospective acceptability to receive advice about cancer prevention, awareness and early diagnosis and especially about ongoing symptoms. Both cognitive and affective acceptability were relatively high indicating that survey participants were cognizant about the potential benefits of advice for their current and future health. The potential to cause anxiety was a concern and potential barrier to implementation raised by health professionals previously (Evans et al., 2023) yet the current study suggests that although around one fifth of the sample indicated some advice types could cause stress, anxiety or fear, overall the emotional cost did not appear prohibitive.

Respondents preferred advice to be delivered during an appointment with a USC pathway doctor or health care professional, or GP, soon after the USC referral results – consistent with the notion of teachable moments. Current receipt of this type of support appears low, suggesting an unmet need within this large patient group. There was no significant difference in acceptability or willingness to receive advice across three USC referral pathways but there were small but significant differences in both acceptability and willingness to receive advice, between the four different types, with lowest willingness and affective acceptability for advice about spotting signs of early or common cancers. These findings may reflect general fears around cancer (Vrinten et al., 2017, Miles et al., 2008) and may also explain why advice about reducing future risk of cancer was seen as most acceptable.

Exploring associations between acceptability and socio-demographic factors is important to enable planning for equitable intervention development. Promisingly, there were very few associations between socio-demographics (including level of deprivation) and prospective acceptability. However there were some differences lower educational attainment was associated with lower affective and cognitive acceptability and people from ethnic minorities reported lower affective acceptability.

Together our findings suggest that the development of a package of advice that encourages early detection and prevention of future cancer may be acceptable across USC pathways. Future intervention development work should be co-designed with a public and patient involvement group that includes people from different ethnicities and educational backgrounds, using qualitative methods, to optimise the acceptability for all and to avoid worsening existing inequalities in cancer awareness and early diagnosis related to ethnicity and education (Niksic et al., 2016, Damiani et al., 2015). Future research should explore ways to deliver advice that recognises patients’ preferences for timely, clinician-led support. HCPs have described potential barriers (Evans et al., 2023) that may conflict with patient preferences. For example, delays in the communication of USC referral results to primary care, time pressures in both primary care and secondary care, and staff shortages may limit the opportunity to offer personalised timely advice. Post-pandemic, digital communication methods have increased, this contrasts with the patient preferences for in-person communication seen here, potentially arising from lower confidence around technology in this older sample. Developing inclusive cost-effective means to support patients, who may differ in electronic health literacy, is important.

### 4.1 Limitations and strengths

This study was the first survey, to our knowledge, to report the perspectives of patients who are found not to have cancer after USC referral, and used a theoretically-informed measurement of acceptability that assessed both cognitive and emotional responses to advice. Our sample was ethnically diverse and had a higher representation of people from areas of greater deprivation compared to a recent published report of patients assessed as ‘no cancer found’ following USC referral: 25.1 % vs 5.6 % from ethnic minority groups; 49.2 % vs 37.9 % from IMD quintiles 1 and 2 (Scott et al.).

The low survey response rate, potentially due to the length of the questionnaire, the contemporaneous postal strikes in the UK, patients’ reluctance to re-engage in thinking about past health experiences, and lack of participant incentives, limits our confidence about the generalisability of our results. However, we did compare responders with non-responders across a number of variables. Our sample had a relatively high proportion of people reporting education at degree level or above and therefore it is possible that the interest in receiving advice is over-estimated. Participants were older, but reassuringly, age was not found to be associated with acceptability. We did not find significant differences by referral pathway but we only recruited from three cancer pathways and it is possible that experiences and views may be different in other pathways. In addition there may be small differences between pathways that this study was not sufficiently powered to detect. Finally, the survey focused on prospective acceptability i.e. hypothetically asking participants how they might feel if they received advice and support in the future. Whilst this is a useful indicator early in intervention development, it will be important to assess actual acceptability at the point of advice delivery.

## 5 Conclusion

Patients reported positive reactions towards the potential to be given advice after USC when cancer is not found, especially about ongoing symptoms and reducing risk of future cancer.

## Funding acknowledgement

This study was funded by Cancer Research UK (EDDCPJT\100015). This research arises from the CanTest Collaborative, which is funded by Cancer Research UK (C8640/A23385), of which SES is a co-investigator. SES and REE are supported by Barts Charity (G-001520; MRC&U0036).

## CRediT authorship contribution statement

**Ruth E.C. Evans:** Methodology, Investigation, Project administration, Formal analysis, Writing – original draft. **Harriet Watson:** Investigation, Writing – review & editing. **Jo Waller:** Conceptualization, Methodology, Writing – review & editing, Funding acquisition. **Brian D. Nicholson:** Conceptualization, Methodology, Writing – review & editing, Funding acquisition. **Thomas Round:** Conceptualization, Methodology, Writing – review & editing, Funding acquisition. **Carolynn Gildea:** Conceptualization, Methodology, Writing – review & editing, Funding acquisition. **Debs Smith:** Conceptualization, Methodology, Writing – review & editing, Funding acquisition. **Suzanne E. Scott:** Conceptualization, Methodology, Writing – original draft, Supervision, Funding acquisition.

## Data availability

Data will be made available on request at https://doi.org/10.18742/25379683.

## Declaration of competing interest

The authors declare that they have no known competing financial interests or personal relationships that could have appeared to influence the work reported in this paper.
