# Supplementary material for: Advice after urgent suspected cancer referral when cancer is not found in England: Survey of patients’ preferences and perceived acceptability
Source: Prev Med Rep. 2024 Jun 10;43:102781. doi: 10.1016/j.pmedr.2024.102781 (PMC11225704; doi:10.1016/j.pmedr.2024.102781)
Supplement: Supplementary Data 1 [file mmc1.docx]

**SUPPLEMENTARY FILE: Advice after urgent suspected cancer referral when cancer is not found in England: survey of patients’ preferences and perceived acceptability**

[Supplementary Table S1: Comparison of age, gender and time since result between responders and non-responders of a survey sent between October 2022 and January 2023 to patients who had been referred on an urgent suspected cancer pathway and no cancer was found 2](#_Toc167999448)

[Supplementary Table S2: Proportion (count, percentage, confidence intervals) of patients willing to receive advice after urgent suspected cancer referral when no cancer was found who responded to a survey between October 2022 and January 2023, stratified by referral pathway 3](#_Toc167999449)

[Supplementary Table S3: Median (range), mean and standard deviation of measure of acceptability of receiving advice after urgent suspected cancer referral when no cancer was found in patients who responded to a survey between October 2022 and January 2023 4](#_Toc167999450)

[Supplementary Table S4: Proportions (count, percent) of patients who responded to a survey between October 2022 and January 2023 who agreed with acceptability statements about receiving advice after urgent suspected cancer referral when no cancer was found 5](#_Toc167999451)

[Supplementary Table S5: Unadjusted univariate linear regressions to assess association between cognitive acceptability and socio-demographic, health and health behaviour factors of patients who had been referred on an urgent suspected cancer pathway with no cancer found who responded to a survey between October 2022 and January 2023 6](#_Toc167999452)

[Supplementary Table S6: Unadjusted univariate linear regressions to assess association between affective acceptability and socio-demographic, health and health behaviour factors of patients who had been referred on an urgent suspected cancer pathway with no cancer found who responded to a survey between October 2022 and January 2023 8](#_Toc167999453)

[Supplementary Table S7: Multiple regression model between cognitive acceptability and socio-demographic, health and health behaviour factors, by advice type in patients who had been referred on an urgent suspected cancer pathway with no cancer found who responded to a survey between October 2022 and January 2023 10](#_Toc167999454)

[Supplementary Table S8: Multiple regression model between affective acceptability and socio-demographic, health and health behaviour factors, by advice type in patients who had been referred on an urgent suspected cancer pathway with no cancer found who responded to a survey between October 2022 and January 2023 11](#_Toc167999455)

[Supplementary Figure S9: Bar chart of proportion (percentage) of patients who responded to a survey between October 2022 and January 2023 regarding preferences for which health professional should give advice after urgent suspect cancer referral 12](#_Toc167999456)

# **Supplementary Table S1: Comparison of age, gender and time since result between responders and non-responders of a survey sent between October 2022 and January 2023 to patients who had been referred on an urgent suspected cancer pathway and no cancer was found**

|  | **Total Sample**  **N=2541** | **Responder**  **n= 406** | **Non-responder**  **n= 2135** | **Group difference**  **Statistic** |
| --- | --- | --- | --- | --- |
| **Mean Age (sd)** | 59.34(17.23)  17-98 | 62.37(16.04)  17-84 | 58.76 (17.39)  17-98 | t(601)=4.10; p<.001 |
| **Gender:** |  |  |  |  |
| Male | 1077 (42.38) | 177 (43.70) | 900 (42.13) | *X*^2^(1,2541)=.343, p=.558 |
| Female | 1464 (57.76) | 228 (56.30) | 1236 (57.87) |  |
| **Interval**  (between when patients’ informed of no cancer and date questionnaire sent): | 89.96(34.68)  32-160 | 89.99 (35.58)  32-159 | 89.95 (31.25)  32-160 | t(530)=.016, p=.986 |

# **Supplementary Table S2: Proportion (count, percentage, confidence intervals) of patients willing to receive advice after urgent suspected cancer referral when no cancer was found who responded to a survey between October 2022 and January 2023, stratified by referral pathway**

|  | **Dichotomised outcome** | | | | **Pathway Group Difference** |
| --- | --- | --- | --- | --- | --- |
|  |  | **Upper GI**  **(CI)** | **Lower GI**  **(CI)** | **Head & Neck**  **(CI)** |  |
| **Willingness to receive advice about ……** |  |  |  |  |  |
| **Ongoing symptoms** (n=388) |  |  |  |  |  |
| No, definitely not | Not willing | 7.1 | 5.4 | 5.5 | p=0.815* |
| No, probably not |  |  |  |  |  |
| Not sure |  |  |  |  |  |
| Yes, probably | Willing | 92.9 | 94.6 | 94.5 |  |
| Yes, definitely |  |  |  |  |  |
| **Spotting early symptoms of different types of cancer** (n=389) |  |  |  |  |  |
| No, definitely not | Not willing | 8.9 | 10.8 | 13.0 | *X*^2^(2,389)=0.645; p=0.724 |
| No, probably not |  |  |  |  |  |
| Not sure |  |  |  |  |  |
| Yes, probably | Willing | 91.1 | 89.2 | 87.0 |  |
| Yes, definitely |  |  |  |  |  |
| **Cancer screening** (n=379) |  |  |  |  |  |
| No, definitely not | Not willing | 7.5 | 8.4 | 7.9 | p=1.0* |
| No, probably not |  |  |  |  |  |
| Not sure |  |  |  |  |  |
| Yes, probably | Willing | 92.5 | 91.6 | 92.1 |  |
| Yes, definitely |  |  |  |  |  |
| **Reducing risk of future cancer** (n=376) |  |  |  |  |  |
| No, definitely not | Not willing | 5.8 | 6.4 | 11.1 | P=0.342* |
| No, probably not |  |  |  |  |  |
| Not sure |  |  |  |  |  |
| Yes, probably | Willing | 94.2 | 93.6 | 88.9 |  |
| Yes, definitely |  |  |  |  |  |

*Fisher’s exact test used to test for group difference because minimum cells numbers were not met

# **Supplementary Table S3: Median (range), mean and standard deviation of measure of acceptability of receiving advice after urgent suspected cancer referral when no cancer was found in patients who responded to a survey between October 2022 and January 2023**

| **Type of advice** | **Cognitive Acceptability**  **Summed Score Range (8-40)** | | **Affective Acceptability**  **Summed Score Range (5-25)** | |
| --- | --- | --- | --- | --- |
|  | **Median** | **Mean (sd)** | **Median (IQR)** | **Mean (sd)** |
| **Ongoing symptoms** | 33.0 (30-37) | 33.02 (5.13) | 20.0 (17-23) | 19.54 (4.31) |
| **Spotting early symptoms of different types of cancer** | 32.0 (30-37 | 32.75 (5.31) | 20.0 (16-22) | 19.10 (4.36) |
| **Cancer Screening** | 32.0 (30-38) | 32.91 (5.43) | 20.0 (17-24) | 19.76 (4.27) |
| **Reducing risk of future cancer** | 33.0 (30-36) | 32.78 (5.18) | 20.0 (18-25) | 20.18 (4.14) |

# **Supplementary Table S4: Proportions (count, percent) of patients who responded to a survey between October 2022 and January 2023 who agreed with acceptability statements about receiving advice after urgent suspected cancer referral when no cancer was found**

| Receiving advice about …..  …..sometime after my urgent referral appointment... | **% (total n) respondents answering agree and strongly agree combined** | | | | | | | |
| --- | --- | --- | --- | --- | --- | --- | --- | --- |
|  | **Ongoing Symptoms** | | **Spotting early cancer symptoms** | | **Cancer Screening** | | **Reducing risk of future cancer** | |
| **Cognitive acceptability (8 scale items)** |  |  |  |  |  |  |  |  |
| *...would have reassured me* | 82.1 | (375) | 74.8 | (369) | 77.2 | (364) | 74.5 | (365) |
| *...would have taken too much time* | 6.5 | (367) | 7.1 | (366) | 5.6 | (359) | 3.9 | (357) |
| *...has benefits that outweigh the costs ** | 75.1 | (365) | 76.6 | (367) | 75.6 | (360) | 73.6 | (356) |
| *...would not have been a priority for me* | 15.2 | (369) | 18.0 | (367) | 18.6 | (360) | 22.8 | (356) |
| *...is important* | 87.9 | (371) | 87.1 | (373) | 89.3 | (364) | 90.0 | (360) |
| *...is a good idea* | 90.6 | (371) | 87.2 | (368) | 90.0 | (360) | 90.3 | (360) |
| *..makes sense to me* | 89.2 | (370) | 87.8 | (369) | 87.5 | (360) | 89.4 | (358) |
| *..would help me know when to contact the doctor with symptoms* | 89.8 | (371) | 91.1 | (369) | - | - | - | - |
| *..would make me more likely to take part in*  *cancer screening* | - | - | - | - | 83.4 | (362) | - | - |
| *..would make me more likely to make changes to reduce my chances of developing cancer* | - | - | - | - | - | - | 84.7 | (360) |
| **Affective acceptability (5 scale items)** |  |  |  |  |  |  |  |  |
| *...would have made me feel anxious* | 24.7 | (365) | 27.0 | (370) | 21.2 | (363) | 17.3 | (359) |
| *...would have made me feel judged* | 5.8 | (361) | 4.7 | (363) | 4.5 | (358) | 2.6 | (352) |
| *...would have made me fearful* | 17.2 | (360) | 20.9 | (369) | 16.1 | (361) | 13.6 | (353) |
| *...would have made me feel embarrassed* | 5.8 | (359) | 4.1 | (363) | 3.3 | (359) | 2.3 | (353) |
| *...would have been stressful for me* | 18.0 | (361) | 19.7 | (366) | 17.5 | (361) | 15.9 | (353) |
| ** benefits/costs to you or the NHS* |  |  |  |  |  |  |  |  |

# **Supplementary Table S5: Unadjusted univariate linear regressions to assess association between cognitive acceptability and socio-demographic, health and health behaviour factors of patients who had been referred on an urgent suspected cancer pathway with no cancer found who responded to a survey between October 2022 and January 2023**

|  | **Variable** | **Cognitive Acceptability** | | | | | | | | | | | | | | | |
| --- | --- | --- | --- | --- | --- | --- | --- | --- | --- | --- | --- | --- | --- | --- | --- | --- | --- |
|  |  | **Ongoing Symptoms** | | | | **Spotting early cancer symptoms** | | | | **Cancer Screening** | | | | **Reducing risk of future cancer** | | | |
|  |  | B (SE) | CI | t | p | B (SE) | CI | t | p | B (SE) | CI | t | p | B (SE) | CI | t | p |
| **SOCIO-DEMOGRAPHIC** | |  |  |  |  |  |  |  |  |  |  |  |  |  |  |  |  |
| **Age** | | -.042  (.018) | -.077 - - .006 | -2.301 | .022 | -.037  (.019) | -.074 - 0.000 | -1.950 | .052 | -.048  (.019) | -.086 - -.009 | -2.450 | .015 | -.050  (.019) | -.087 - -.013 | -2.669 | .008 |
| **Pathway** | *^(ref)^Lower GI* | - | - | - | - | - | - | - | - | - | - | - | - | - | - | - | - |
|  | *Upper GI* | -1.874 (.804) | -3.454 - -.293 | -2.332 | .020 | -.843 (.823) | -2.462 -.776 | -1.023 | .307 | -.220  (.864) | -1.920 -1.479 | -.255 | .799 | -.251 (.841) | -1.904 -1.403 | -.298 | .766 |
|  | *Head and Neck* | .111 (.641) | -1.149 - 1.371 | .173 | .862 | -.243 (.670) | -1.560 -1.074 | -.363 | .717 | -.728  (.692) | -2.089 -.633 | -1.052 | .294 | -.562  (.659) | -1.858 -.733 | -.854 | .394 |
| **Ethnic group** | *^(ref)^White* | - |  |  |  |  |  |  |  |  |  |  |  |  |  |  |  |
|  | *Non-white ethnicity* | -.893  (.635) | -2.142 - .356 | -1.406 | .161 | -1.573  (.660) | -2.871 - -.275 | -2.384 | .018 | -1.394  (.691) | -2.754 - -.034 | -2.017 | .045 | -.479  (.659) | -1.775 - .817 | -.727 | .468 |
| **Marital status** | *^(ref)^Single* | - | - | - | - | - | - | - | - | - | - | - | - | - | - | - | - |
|  | *Married/ civil partnership* | -.355  (.628) | -1.589 - .880 | -.565 | .572 | -1.159  (.651) | -2.440 - .122 | -1.779 | .076 | -.966  (.678) | -2.30 -.369 | -1.423 | .156 | -.392  (.649) | -1.668 - 0.884 | -.604 | .546 |
|  | *Separated/ divorced* | -1.153  (1.116) | -3.347 - 1.042 | 1.033 | .302 | -2.667  (1.121) | -4.872 --.462 | -2.379 | 0.018 | -.927  (1.180) | -3.248 - 1.393 | -.786 | .432 | -1.060  (1.145) | -3.312 - 1.192 | -.926 | .355 |
|  | *Widowed* | -1.242  (1.099) | -3.405 - .920 | -1.130 | .259 | -2.710  (1.106) | -4.885 - -.534 | -2.450 | 0.015 | -2.150  (1.180) | -4.470 - 0.171 | -1.822 | .069 | -.923  (1.112) | -3.110 - 1.265 | -.830 | .407 |
| **Education** | *^(ref)^None, GCSE, Vocational* | - | - | - | - | - | - | - | - | - | - | - | - | - | - | - | - |
|  | *A level or equiv.* | 1.933  (.873) | .216 - 3.650 | 2.214 | .027 | .318  (.924) | -1.500 -2.136 | .344 | .731 | .911  (.940) | -.938 -2.760 | .969 | .333 | 1.149  (.891) | -.604 -2.901 | 1.289 | .198 |
|  | *Higher Education* | 2.731  (.580) | .591 - 3.871 | 4.712 | <.001 | 1.971  (.617) | .763 -- 3.191 | 3.203 | <.001 | 2.284  (.631) | 1.042 -3.526 | 3.618 | <.001 | 2.811  (.599) | 1.633-3.989 | 4.694 | <.001 |
| **IMD** | *^(ref)^Higher (1-5)* | - | - | - | - | - | - | - | - | - | - | - | - | - | - | - | - |
|  | *Lower (6-10)* | -.370  (.656) | -1.661 - .0922 | -.563 | .574 | -.550  (.660) | -1.849 - .750 | -.833 | .406 | -.537  (.671) | -1.859 - .784 | -.800 | .424 | .168  (.610) | -1.033 -1.369 | 0.275 | .784 |
| **HEALTH** | |  |  |  |  |  |  |  |  |  |  |  |  |  |  |  |  |
| **Cancer diagnosis** | *^(ref)^No* | - | - | - | - | - | - | - | - | - | - | - | - | - | - | - | - |
|  | *Yes* | -.743  (.722) | -2.163 - .677 | -1.029 | .304 | -.392  (.749) | -1.866 -1.082 | -.523 | .602 | .503  (.770) | -1.012 - 2.018 | .653 | .514 | -.458  (.745) | -1.923 - 1.007 | -.615 | .539 |
| **Any co-morbidity** | *^(ref)^No* | - | - | - | - | - | - | - | - | - | - | - | - | - | - | - | - |
|  | *Yes* | -.724 (.633) | -1.968 - .520 | -1.144 | .253 | -.438  (.663) | -1.742 - .866 | -.660 | .510 | -.540  (.683) | -1.884 -.803 | -.791 | .430 | -.758  (.661) | -2.058 -.541 | -1.148 | .252 |
| **HEALTH BEHAVIOUR** | |  |  |  |  |  |  |  |  |  |  |  |  |  |  |  |  |
| **Bowel screening in last 2 years** | *^(Ref)^No* | - | - | - | - | - | - | - | - | - | - | - | - | - | - | - | - |
|  | *Yes* | .817  (.688) | -.537 - 2.170 | 1.187 | .236 | 1.086  (.708) | -.306 - 2.479 | 1.534 | .126 | 1.057  (.746) | -.411 - 2.524 | 1.416 | .158 | .839  (.695) | -.527 -2.206 | 1.208 | .228 |
| **GP visits in last year** |  | -.057  (.071) | -.197 - .083 | -.807 | .420 | .062  (.074) | -.083 - .207 | .840 | .402 | .034  (.078) | -.120 - .188 | .430 | .668 | .005  (.074) | -.141 - .151 | .063 | .950 |
| **Fruit and vegetable consumption** | *^(Ref)^Not met guidelines* | - | - | - | - | - | - | - | - | - | - | - | - | - | - | - | - |
|  | *Met guidelines* | 1.259  (.631) | .018 - 2.499 | 1.995 | .047 | 1.313  (.644) | .046 -2.580 | 2.037 | .042 | 1.919*  (.659) | .623 -3.216 | 2.912 | .004 | 1.781*  (.629) | .544 -3.018 | 2.831 | .005 |
| **Bowel screening in last 2 years** | *^(Ref)^No* | - | - | - | - | - | - | - | - | - | - | - | - | - | - | - | - |
|  | *Yes* | .817  (.688) | -.537 - 2.170 | 1.187 | .236 | 1.086  (.708) | -.306 - 2.479 | 1.534 | .126 | 1.057  (.746) | -.411 - 2.524 | 1.416 | .158 | .839  (.695) | -.527 -2.206 | 1.208 | .228 |
| **Smoking** | *^(Ref)^Never smoked* | - | - | - | - | - | - | - | - | - | - | - | - | - | - | - | - |
|  | *Used to smoke/ current smoker* | .957  (.548) | -.120 - 2.034 | 1.747 | .081 | .626  (.563) | -.481 - 1.733 | 1.112 | .267 | .233  (.582) | -.913 - 1.379 | .400 | .689 | -.189  (.557) | -1.284 - .906 | -.340 | .734 |
| **Exercise** | | .138  (.120) | -.099 - .375 | 1.148 | .252 | .119  (.125) | -.126 - .364 | .955 | .340 | .185  (.128) | .067 -.437 | 1.444 | .150 | .258  (.122) | -.018 -.497 | 2.117 | .035 |
| **Alcohol** | *^(ref)^ Audit C <5* | - | - | - | - | - | - | - | - | - | - | - | - | - | - | - | - |
|  | *Audit C >5* | .377  (.654) | -.909 - 1.663 | .576 | .565 | .209  (.661) | -1.092 - 1.509 | .315 | .753 | .239  (.688) | -1.114 - 1.592 | .347 | .729 | -.483 (.655) | -1.771 - .806 | -.737 | .462 |

# **Supplementary Table S6: Unadjusted univariate linear regressions to assess association between affective acceptability and socio-demographic, health and health behaviour factors of patients who had been referred on an urgent suspected cancer pathway with no cancer found who responded to a survey between October 2022 and January 2023**

| Variable | | **Affective Acceptability** | | | | | | | | | | | | | | | |
| --- | --- | --- | --- | --- | --- | --- | --- | --- | --- | --- | --- | --- | --- | --- | --- | --- | --- |
|  |  | **Ongoing Symptoms** | | | | **Spotting early cancer symptoms** | | | | **Cancer Screening** | | | | **Reducing risk of future cancer** | | | |
|  |  | B (SE) | CI | t | p | B (SE) | CI | t | p | B (SE) | CI | t | p | B (SE) | CI | t | p |
| **SOCIO-DEMOGRAPHIC** | |  |  |  |  |  |  |  |  |  |  |  |  |  |  |  |  |
| **Age** | | -.033  (.015) | -.061 - -.004 | -2.223 | .027 | -.035  (.015) | -.063 - -.006 | -2.374 | .018 | -.029  (.015) | -.058 - -.001 | -2.032 | .043 | -.014  (.014) | -.042 - .014 | -.996 | .320 |
| **Pathway** | *^(ref)^Lower GI* | - | - | - | - | - | - | - | - | - | - | - | - | - | - | - | - |
|  | *Upper GI* | -.912  (.678) | -2.246 - .422 | -1.345 | .180 | -1.275  (.665) | -2.583 - .033 | -1.917 | .056 | -1.009  (.677) | -2.341 -.322 | -1.491 | .137 | -.254  (.671) | -1.575 - 1.066 | -.379 | .705 |
|  | *Head and Neck* | .423  (.546) | -.650 - 1.497 | .776 | .438 | .792  (.550) | -.290 - 1.874 | 1.440 | .151 | .390  (.544) | -.681 -1.460 | .716 | .475 | .566  (.535) | -.487 - 1.619 | 1.057 | .291 |
| **Ethnic group** | *^(ref)^White* | - |  |  |  |  |  |  |  |  |  |  |  |  |  |  |  |
|  | *Other ethnic groups* | -1.920  (.534) | -2.971 - -.869 | -3.594 | <.001 | -2.187  (.532) | -3.234 --1.141 | -4.110 | <.001 | -2.631  (.524) | -3.661- -1.601 | -5.024 | <.001 | -1.308  (0.524) | -2.339 - -.276 | -2.494 | .013 |
| **Marital status** | *^(ref)^Single* | - | - | - | - | - | - | - | - | - | - | - | - | - | - | - | - |
|  | *Married/ civil partnership* | -.151  (.526) | -1.185 - .884 | -.286 | .775 | -.670  (.532) | -1.717 - .377 | -1.260 | .209 | -.249  (.517) | -1.267 - .769 | -.481 | .631 | -.194  (.505) | -1.187 - .799 | -.384 | .701 |
|  | *Separated/ divorced* | -2.319  (.946) | -4.180 - -.459 | -2.452 | .015 | -1.530  (.925) | -3.350 - .291 | -1.653 | .099 | -2.024  (.885) | -3.764 - -.283 | -2.287 | .023 | -2.766  (.874) | -4.484- -1.047 | -3.166 | .002 |
|  | *Widowed* | -.421  (.931) | -2.252 - 1.411 | -.452 | .652 | -1.096  (.889) | -2.845 - .652 | -1.234 | .218 | .242  (.897) | -1.523 - 2.008 | .270 | .787 | -.227  (.861) | -1.922 - 1.467 | -.264 | .792 |
| **Education** | *^(ref)^None, GCSE, Vocational* | - | - | - | - | - | - | - | - | - | - | - | - | - | - | - | - |
|  | *A level or equiv.* | .577  (.744) | -.887 - 2.040 | .775 | .439 | .109  (.747) | -1.360 - 1.577 | .145 | .884 | .227  (.722) | -1.192 - 1.647 | 0.315 | .753 | .048  (.708) | -1.345 - 1.442 | .068 | .946 |
|  | *Higher Education* | 2.024  (.492) | 1.056 - 2.993 | 4.113 | <.001 | 1.456  (.489) | .494 - 2.419 | .2.976 | .003 | 1.985  (.475) | 1.050 - 2.920 | 4.176 | <.001 | 2.060  (.468) | 1.140 - 2.981 | 4.402 | <.001 |
| **Level of deprivation** | *^(ref)^higher (1-5)* | - | - | - | - | - | - | - | - | - | - | - | - | - | - | - | - |
|  | *Lower (6-10)* | .213  (.531) | -.832 - 1.259 | .402 | .688 | -.007  (.531) | -1.053 - 1.039 | -0.013 | .989 | -0.013  (.519) | -1.034 - 1.008 | -0.026 | .979 | .588  (.516) | -0.428 - 1.605 | 1.140 | .255 |
| **HEALTH** | |  |  |  |  |  |  |  |  |  |  |  |  |  |  |  |  |
| **Cancer diagnosis** | *^(ref)^No* | - | - | - | - | - | - | - | - | - | - | - | - | - | - | - | - |
|  | *Yes* | -.501  (.594) | -1.688 - .667 | -.843 | .400 | -.114  (.602) | -1.297 - 1.070 | -.189 | .850 | 0.292  (.590) | -.868 - 1.451 | .495 | .621 | .607  (.586) | -.546 - 1.759 | 1.036 | .301 |
| **Any co-morbidity** | *^(ref)^No* |  |  |  |  |  |  |  |  |  |  |  |  |  |  |  |  |
|  | *Yes* | .988  (.533) | -.060 - 2.036 | 1.855 | .064 | .636  (.544) | -.434 - 1.706 | 1.168 | .244 | .697  (.536) | -.358 - 1.752 | 1.30 | .194 | 0.933  (.528) | -.106 - 1.971 | 1.767 | .078 |
| **HEALTH BEHAVIOURS** | |  |  |  |  |  |  |  |  |  |  |  |  |  |  |  |  |
| **Bowel screening in last 2 years** | *^(Ref)^No* | - | - | - | - | - | - | - | - | - | - | - | - | - | - | - | - |
|  | *Yes* | .518  (.571) | -.605 - 1.642 | .908 | .365 | .573  (.574) | -.557 - 1.703 | .998 | .319 | .649  (.556) | -.445 - 1.743 | 1.167 | .244 | .753  (.550) | -.329 - 1.836 | 1.369 | .172 |
| **GP visits in last year** | | -.023  (.059) | -.140 - .094 | -.390 | .697 | .052  (.059) | -.064 - .168 | 0.886 | .376 | -.027  (.058) | -.141 - .088 | -.456 | .649 | -.025  (.058) | -.138 - .089 | -.424 | .672 |
| **Fruit and vegetable consumption** | *^(Ref)^Not met guidelines* | - | - | - | - | - | - | - | - | - | - | - | - | - | - | - | - |
|  | *Met guidelines* | 1.240  (.514) | .229 - 2.252 | 2.413 | .016 | .995  (.514) | -.017 - 2.007 | 1.934 | .054 | 1.363  (.499) | .382 - 2.344 | 2.733 | .007 | 1.053  (.496) | .078 - 2.028 | 2.124 | .034 |
| **Smoking** | *^(Ref)^Never smoked* | - | - | - | - | - | - | - | - | - | - | - | - | - | - | - | - |
|  | *Used to/ or Current smoker* | .936  (.454) | .043 - 1.828 | 2.061 | .040 | .670  (.452) | -.219 - 1.559 | 1.482 | .139 | .953  (.442) | .084 - 1.822 | 2.157 | .032 | .435  (.437) | -.425 - 1.294 | .994 | .321 |
| **Exercise frequency** | | .124  (.10) | -.072 - .320 | 1.246 | .214 | .183  (.099) | -.012 - .378 | 1.848 | .065 | .195  (.097) | .004 - .387 | 2.005 | .046 | .162  (.096) | -.026 - .351 | 1.692 | .092 |
| **Alcohol use** | *^(ref)^ Audit C <5* | - | - | - | - | - | - | - | - | - | - | - | - | - | - | - | - |
|  | *Audit C >5* | 1.069  (.532) | .023 - 2.115 | 2.010 | .045 | 1.065  (.540) | .004 - 2.126 | 1.973 | .049 | 0.768  .528) | -.271 - 1.807 | 1.454 | .147 | .467  (.523) | -.561 - 1.496 | .894 | .372 |

# **Supplementary Table S7: Multiple regression model between cognitive acceptability and socio-demographic, health and health behaviour factors, by advice type in patients who had been referred on an urgent suspected cancer pathway with no cancer found who responded to a survey between October 2022 and January 2023**

| **Variable** | | **Cognitive Acceptability** | | | | | | | | | | | | | | | |
| --- | --- | --- | --- | --- | --- | --- | --- | --- | --- | --- | --- | --- | --- | --- | --- | --- | --- |
|  |  | **Ongoing Symptoms** | | | | **Spotting early cancer symptoms** | | | | **Cancer Screening** | | | | **Reducing risk of future cancer** | | | |
|  |  | **B (SE)** | **CI** | **t** | **p** | **B (SE)** | **CI** | **t** | **p** | **B (SE)** | **CI** | **t** | **p** | **B (SE)** | **CI** | **t** | **p** |
| **Age** |  | -.015 (.022) | -.057 - .028 | -.674 | .501 | -.019 (.024) | -.066 - .027 | -.813 | .417 | -.045 (.025) | -.093 - .004 | -1.810 | .071 | -.044 (.023) | -.089 - .001 | -1.906 | .058 |
| **Pathway** | *^(ref)^Lower GI* |  |  |  |  |  |  |  |  |  |  |  |  |  |  |  |  |
|  | *Upper GI* | -1.932 (.898) | - 3.700--.164 | -2.152 | .032 | -.896 (.968) | -2.801-1.008 | -.926 | .355 | .111 (.995) | - 1.847-2.070 | .112 | .911 | -.302 (.950) | - 2.173-1.569 | -.318 | .751 |
|  | *Head and Neck* | -.674 (.724) | -2.100- .751 | -.931 | .353 | -.966 (.801) | -2.542- .610 | -1.206 | .229 | -1.507 (.831) | -3.143- .130 | -1.812 | .071 | -1.186 (.768) | -2.698- .326 | -1.544 | .124 |
| **Ethnic group** | *^(ref)^White* |  |  |  |  |  |  |  |  |  |  |  |  |  |  |  |  |
|  | *Other ethnic groups* | -.967 (.712) | -2.368 - .435 | -1.357 | .176 | -1.551 (.788) | -3.102 - -.001 | -1.970 | .050 | -1.525 (.825) | -3.149 - .099 | -1.849 | .066 | -.549 (.762) | -2.049 - .950 | -.721 | .471 |
| **Marital status** | *^(ref)^Single* |  |  |  |  |  |  |  |  |  |  |  |  |  |  |  |  |
|  | *Married/ civil partnership* | -.061 (.687) | -1.413 - 1.290 | -.089 | .929 | -1.031 (.761) | -2.530 - .467 | -1.355 | .177 | -.612 (.786) | -2.160 - .936 | -.778 | .437 | .110 (.742) | -1.350 - 1.570 | .148 | .882 |
|  | *Separated/ divorced* | -.566 (1.291) | -3.107 - 1.975 | -.439 | .661 | -2.618 (1.392) | -5.358 - .121 | -1.881 | .061 | -.345 (1.410) | -3.120 - 2.431 | -.245 | .807 | -.351 (1.352) | -3.012 - 2.310 | -.260 | .795 |
|  | *Widowed* | -.645 (1.257) | -3.119 - 1.829 | -.513 | .608 | -2.360 (1.345) | -5.009 - .288 | -1.754 | .080 | -1.579 (1.407) | -4.348 - 1.191 | -1.122 | .263 | .087 (1.304) | -2.480 - 2.655 | .067 | .947 |
| **Education** | *^(ref)^None, GCSE, Vocational* |  |  |  |  |  |  |  |  |  |  |  |  |  |  |  |  |
|  | *A level or equiv.* | 2.014 ( 1.010) | .025 - 4.003 | 1.993 | .047 | .474 (1.081) | -1.654 - 2.603 | .439 | .661 | .713 (1.112) | -1.475 - 2.901 | .642 | .522 | 1.211 (1.049) | -.854 - 3.276 | 1.155 | .249 |
|  | *Degree Educated* | 2.568 ( .674) | 1.242 - 3.894 | 3.813 | <.001 | 1.536 (.741) | .077 - 2.995 | 2.072 | .039 | 1.591 (.765) | .086 - 3.097 | 2.080 | .038 | 2.476 (.720) | 1.059 - 3.893 | 3.440 | <.001 |
| **Fruit and vegetable consumption** | *^(Ref)^Not met guidelines* |  |  |  |  |  |  |  |  |  |  |  |  |  |  |  |  |
|  | *Met guidelines* | .281 (.700) | -1.098 - 1.660 | .402 | .688 | .601 (.760) | -.895 - 2.097 | .790 | .430 | 1.061 (.783) | -.480 - 2.603 | 1.355 | .176 | .642 (.739) | -.811 - 2.096 | .870 | .385 |
| **Exercise** |  | .004(.131) | -.254 - .262 | .031 | .976 | -.020 (.142) | -.301 - .260 | -.143 | .886 | .051 (.147) | -.239 - .340 | .344 | .731 | .168 (.139) | -.105 - .442 | 1.212 | .226 |
| **Smoking** | *^(Ref)^Never smoked* |  |  |  |  |  |  |  |  |  |  |  |  |  |  |  |  |
|  | *Used to/ currently smoke* | .741 (.633) | -.506 - 1.988 | 1.170 | .243 | .570 (.689) | -.786 - 1.927 | .827 | .409 | .034 (.708) | -1.360 - 1.428 | .048 | .962 | .269 (.665) | -1.039 - 1.578 | .405 | .685 |
| **Alcohol** | *^(ref)^* Audit C <*5* |  |  |  |  |  |  |  |  |  |  |  |  |  |  |  |  |
|  | *Audit C >5* | -.353 (.723) | -1.776 - 1.070 | -.488 | .626 | -.292 (.777) | -1.821 - 1.237 | -.375 | .708 | -.029 (.801) | -1.605 - 1.548 | -.036 | .972 | -.918 (.753) | -2.401 - .565 | -1.219 | .224 |
| ***Adjusted R2*** |  | .054 | | | | .035 | | | | .035 | | | | .058 | | | |
| ***F*** |  | 2.277 | | | | 1.810 | | | | 1.814 | | | | 2.377 | | | |
| ***df*** |  | 276 | | | | 280 | | | | 278 | | | | 279 | | | |
| ***p*** |  | .007 | | | | .041 | | | | .041 | | | | .005 | | | |

# **Supplementary Table S8: Multiple regression model between affective acceptability and socio-demographic, health and health behaviour factors, by advice type in patients who had been referred on an urgent suspected cancer pathway with no cancer found who responded to a survey between October 2022 and January 2023**

| **Variable** | | **Affective Acceptability** | | | | | | | | | | | | | | | | |
| --- | --- | --- | --- | --- | --- | --- | --- | --- | --- | --- | --- | --- | --- | --- | --- | --- | --- | --- |
|  |  | **Ongoing Symptoms** | | | | **Spotting early cancer symptoms** | | | | **Cancer Screening** | | | | | **Reducing risk of future cancer** | | | |
|  |  | **B (SE)** | **CI** | **t** | **p** | **B (SE)** | **CI** | **t** | **p** | **B (SE)** | **CI** | **t** | **p** | **B (SE)** | | **CI** | **t** | **p** |
| **Age** |  | -.020 (.018) | -.058 -.011 | -1.338 | .182 | -.023 (.018) | -.059-.012 | -1.301 | .194 | -.023 (.017) | -.056-.011 | -1.327 | .185 | .008 (.017) | | -.026-.042 | .471 | .638 |
| **Pathway** | *^(ref)^Lower GI* |  |  |  |  |  |  |  |  |  |  |  |  |  | |  |  |  |
|  | *Upper GI* | -1.558 (.723) | -2.982 - -.134 | -2.154 | .032 | -1.794 (.725) | -3.222 - .366 | -2.473 | .014 | -1.689 (.689) | -3.046--.332 | -2.450 | .015 | -1.152 (.708) | | -2.546-.241 | -1.628 | .105 |
|  | *Head and Neck* | -.365 (.594.) | -1.534 - .804 | -.615 | .539 | -.003 (.606) | -1.196-1.189 | -.005 | .996 | -.404 (.582) | -1.550-.743 | -.693 | .489 | -.304 (.581) | | -1.449-.841 | -.523 | .602 |
| **Ethnic group** | *^(ref)^White* |  |  |  |  |  |  |  |  |  |  |  |  |  | |  |  |  |
|  | *Other ethnic groups* | -1.666 (.582) | -2.812- -.520 | -2.861 | .005 | -1.689 (.593) | -2.856- -.523 | -2.851 | .005 | -2.125 (.571) | -3.250- -1.000 | -3.718 | <.001 | -1.049 (.575) | | -2.181-.083 | -1.824 | .069 |
| **Marital status** | *^(ref)^Single* |  |  |  |  |  |  |  |  |  |  |  |  |  | |  |  |  |
|  | *Married/ civil partnership* | .453 (.555) | -.640-1.547 | .816 | .415 | -.166 (.573) | -1.295-.963 | -.290 | .772 | .491 (.548) | -.589-1.570 | .895 | .371 | .064 (.555) | | -1.029-1.156 | .115 | .909 |
|  | *Separated/ divorced* | -2.654 (1.057) | -4.735- -.573 | -2.511 | .013 | -1.044 (1.043) | -3.097-1.009 | -1.001 | .318 | -1.472 (.979) | -3.399-.455 | -1.504 | .134 | -2.220 (1.007) | | -4.202- -.238 | -2.205 | .028 |
|  | *Widowed* | .311 (1.032) | -1.722- 2.343 | .301 | .764 | -.300 (1.009) | -2.286-1.686 | -.297 | .767 | .597 (.975) | -1.323-2.516 | .612 | .541 | -.327 (.972) | | -2.240-1.587 | -.336 | .737 |
| **Education** | *^(ref)^None, GCSE, Vocational* |  |  |  |  |  |  |  |  |  |  |  |  |  | |  |  |  |
|  | *A level or equiv.* | .727 (.824) | -.894-2.349 | .883 | .378 | -.093 (.826) | -1.719-1.534 | -.112 | .911 | .388 (.786) | -1.159-1.936 | .494 | .622 | .252 (.796) | | -1.316-1.820 | .316 | .752 |
|  | *Degree Educated* | 1.463 (.548) | .385-2.541 | 2.672 | .008 | .587 (.555) | -.507-1.680 | 1.056 | .292 | 1.420 (.530) | .378- 2.463 | 2.681 | .008 | 1.690 (.537) | | .634-2.746 | 3.149 | .002 |
| **Fruit and vegetable consumption** | *^(Ref)^Not met guidelines* |  |  |  |  |  |  |  |  |  |  |  |  |  | |  |  |  |
|  | *Met guidelines* | .470 (.566) | -.644-1.584 | .830 | .407 | .679 (.573) | -.449-1.807 | 1.185 | .237 | .590 (.544) | -.482-1.661 | 1.084 | .279 | .457 (.553) | | -.632-1.546 | .826 | .409 |
| **Exercise** |  | -.022 (.106) | -.231-.187 | -.206 | .837 | .026 (.107) | -.184-.237 | .247 | .805 | .083 (.102) | -.117-.284 | .818 | .414 | .049 (.104) | | -.155-.253 | .472 | .637 |
| **Smoking** | *^(Ref)^Never smoked* |  |  |  |  |  |  |  |  |  |  |  |  |  | |  |  |  |
|  | *Used to smoke / current smoker* | .809 (.515) | -.206 - 1.823 | 1.569 | .118 | .572 (.517) | -.446-1.589 | 1.106 | .270 | .944 (.493) | -.026-1.914 | 1.915 | .057 | .616 (.497) | | -.363-1.595 | 1.239 | .216 |
| **Alcohol** | *^(ref)^* Audit C <*5* |  |  |  |  |  |  |  |  |  |  |  |  |  | |  |  |  |
|  | *Audit C >5* | .352 (.584) | -.798 - 1.501 | .602 | .548 | .299 (.585) | -.854-1.451 | .510 | .610 | -.232 (.556) | -1.327-.863 | -.417 | .677 | -.271 (.562) | | -1.378-.836 | -.482 | .631 |
| ***Adjusted R2*** |  | .099 | | | | .062 | | | | .120 | | | | | .063 | | | |
| ***F*** |  | 3.393 | | | | 2.482 | | | | 4.019 | | | | | 2.500 | | | |
| ***df*** |  | 270 | | | | 277 | | | | 276 | | | | | 276 | | | |
| ***p*** |  | <.001 | | | | .003 | | | | <.001 | | | | | .003 | | | |

# **Supplementary Figure S9: Bar chart of proportion (percentage) of patients who responded to a survey between October 2022 and January 2023 regarding preferences for which health professional should give advice after urgent suspect cancer referral**
