# Supplementary material for: Advice after urgent suspected cancer referral when cancer is not found in England: Survey of patients’ preferences and perceived acceptability
Source: Prev Med Rep. 2024 Jun 10;43:102781. doi: 10.1016/j.pmedr.2024.102781 (PMC11225704; doi:10.1016/j.pmedr.2024.102781)
Supplement: Supplementary Data 2 [file mmc2.pdf]

NHS
Guy's and St Thomas'
NHS Foundation Trust

King's College London

Opportunities to Raise Cancer Awareness After Referral: Survey of Patients' Views

Thank you for agreeing to take part in our research study.

The information you give in this survey will be treated in confidence.

In Section A, the survey describes 4 different types of advice that could be given sometime after someone has had an urgent referral to be seen by hospital specialists and cancer has **not** been found. We would like to hear your views on each type of advice. In Section B, we ask for information about yourself. This will be used to learn how views differ between people.

If you have any questions about this survey, or need help completing it, please contact: Ruth Evans (email: **ruth.e.evans@kcl.ac.uk** or telephone: **0784 0800 309**).

If you would prefer to complete this survey online, scan this QR code or visit this website: https://tinyurl.com/ORCAAR-Study

You will need to input this participant number:

Funded by
CANCER RESEARCH UK

CanTest
Right place, right time, by your family doctor

Section A: Your views on four types of advice

1. Your views on getting advice about **ongoing symptoms**, sometime after having an urgent referral appointment with hospital specialists and cancer has not been found.

1a Would you have been willing to receive advice about **ongoing symptoms**, sometime after your urgent referral appointment?

No, definitely not	No, probably not	Not sure	Yes, probably	Yes, definitely
❑	❑	❑	❑	❑

1b Which of the following would you want to receive advice about?	Yes, I would want to receive advice about this	No, I would not want to receive advice about this	Not applicable
When to contact a doctor or other healthcare professional if symptoms don't go away	❑	❑	❑
What to do about new symptoms	❑	❑	❑
What to do if symptoms get worse	❑	❑	❑
How to manage ongoing symptoms	❑	❑	❑
Who to contact about ongoing symptoms	❑	❑	❑
How to contact someone about ongoing symptoms	❑	❑	❑
Why it is important to get advice about ongoing symptoms	❑	❑	❑
Anything else (please add)………..			

1c Receiving advice about **ongoing symptoms**, sometime after my urgent referral appointment...	Strongly disagree	Disagree	Neither agree or disagree	Agree	Strongly agree
...would have made me feel anxious	❑	❑	❑	❑	❑
...would have made me feel judged	❑	❑	❑	❑	❑
...would have made me fearful	❑	❑	❑	❑	❑
...would have made me feel embarrassed	❑	❑	❑	❑	❑
...would have taken too much time	❑	❑	❑	❑	❑
...would have reassured me	❑	❑	❑	❑	❑
...has benefits that outweigh the costs *	❑	❑	❑	❑	❑
...would not have been a priority for me	❑	❑	❑	❑	❑
...would have been stressful for me	❑	❑	❑	❑	❑
...is important	❑	❑	❑	❑	❑
...is a good idea	❑	❑	❑	❑	❑
...would help me know when to contact the doctor with symptoms	❑	❑	❑	❑	❑
...makes sense to me	❑	❑	❑	❑	❑

* benefits/costs to you or the NHS

1d	When would you have liked to receive advice about **ongoing symptoms**?	*(Tick **Yes** or **No** for each option)*	Yes	No
		With my urgent referral results	❑	❑
		2-4 weeks after receiving my results	❑	❑
		1-3 months after receiving my results	❑	❑
		More than 3 months after receiving my results	❑	❑
		Not at all	❑	❑

1e	How would you have liked to receive advice about **ongoing symptoms**?	*(Tick **Yes** or **No** for each option)*	Yes	No
		Text message with links to videos and/or websites	❑	❑
		Text message chat with a healthcare professional	❑	❑
		In an email	❑	❑
		Leaflet	❑	❑
		Group session with other patients	❑	❑
		In a phone app	❑	❑
		Phone appointment with a healthcare professional	❑	❑
		Video appointment with a healthcare professional	❑	❑
		Face to face appointment with a healthcare professional	❑	❑
		Other (please specify)		

1f	Who would you have liked to give you advice about **ongoing symptoms**?	*(Tick **Yes** or **No** for each option)*	Yes	No
		A doctor from my GP practice	❑	❑
		A nurse or other healthcare professional from my GP practice	❑	❑
		A doctor from the urgent referral clinic	❑	❑
		A nurse or other healthcare professional from the urgent referral clinic	❑	❑
		Somebody else (please specify)		

1g	Have you received any of the following advice about **ongoing symptoms**, since your urgent referral appointment?	Yes	No	Unsure
	When to contact a doctor or other healthcare professional if symptoms don't go away	❑	❑	❑
	What to do about new symptoms	❑	❑	❑
	What to do if symptoms get worse	❑	❑	❑
	How to manage ongoing symptoms	❑	❑	❑
	Who to contact about ongoing symptoms	❑	❑	❑
	How to contact someone about ongoing symptoms	❑	❑	❑
	Why it is important to get advice about ongoing symptoms	❑	❑	❑

2. Your views on getting advice about **spotting early symptoms of different types of cancer**, sometime after having an urgent referral appointment with hospital specialists and cancer has not been found.

2a Would you have been willing to receive advice about spotting **early symptoms of different types of cancer**, sometime after your urgent referral appointment?

No, definitely not	No, probably not	Not sure	Yes, probably	Yes, definitely
❑	❑	❑	❑	❑

2b Which of the following would you want to receive advice about?	Yes, I would want to receive advice about this	No, I would not want to receive advice about this	Not applicable
Early symptoms of different types of cancer	❑	❑	❑
How to spot changes in your body	❑	❑	❑
Why it is important to seek help quickly for possible signs of cancer	❑	❑	❑
How to seek help for possible signs of cancer	❑	❑	❑
Tackling worries or concerns you might have about getting help	❑	❑	❑
Anything else (please add)…			

2c Receiving advice about **spotting early symptoms of different types of cancer**, sometime after my urgent referral appointment...	Strongly disagree	Disagree	Neither agree or disagree	Agree	Strongly agree
...would have made me feel anxious	❑	❑	❑	❑	❑
...would have made me feel judged	❑	❑	❑	❑	❑
...would have made me fearful	❑	❑	❑	❑	❑
...would have made me feel embarrassed	❑	❑	❑	❑	❑
...would have taken too much time	❑	❑	❑	❑	❑
...would have reassured me	❑	❑	❑	❑	❑
...has benefits that outweigh the costs *	❑	❑	❑	❑	❑
...would not have been a priority for me	❑	❑	❑	❑	❑
...would have been stressful for me	❑	❑	❑	❑	❑
...is important	❑	❑	❑	❑	❑
...is a good idea	❑	❑	❑	❑	❑
...would help me know when to contact the doctor with symptoms	❑	❑	❑	❑	❑
...makes sense to me	❑	❑	❑	❑	❑

* benefits/costs to you or the NHS

2d	When would you have liked to receive advice about **spotting early symptoms of different types of cancer**?	*(Tick **Yes** or **No** for each option)*	Yes	No
		With my urgent referral results	❑	❑
		2-4 weeks after receiving my results	❑	❑
		1-3 months after receiving my results	❑	❑
		More than 3 months after receiving my results	❑	❑
		Not at all	❑	❑

2e	How would you have liked to receive advice about **spotting early symptoms of different types of cancer**?	*(Tick **Yes** or **No** for each option)*	Yes	No
		Text message with links to videos and/or websites	❑	❑
		Text message chat with a healthcare professional	❑	❑
		In an email	❑	❑
		Leaflet	❑	❑
		Group session with other patients	❑	❑
		In a phone app	❑	❑
		Phone appointment with a healthcare professional	❑	❑
		Video appointment with a healthcare professional	❑	❑
		Face to face appointment with a healthcare professional	❑	❑
		Other (please specify)		

2f	Who would you have liked to give you advice about **spotting early symptoms of different types of cancer**?	*(Tick **Yes** or **No** for each option)*	Yes	No
		A doctor from my GP practice	❑	❑
		A nurse or other healthcare professional from my GP practice	❑	❑
		A doctor from the urgent referral clinic	❑	❑
		A nurse or other healthcare professional from the urgent referral clinic	❑	❑
		Somebody else (please specify)		

2g	Have you received any of the following advice about **spotting early symptoms of different types of cancer**, since your urgent referral appointment?	Yes	No	Unsure
	Early symptoms of different types of cancer	❑	❑	❑
	How to spot changes in your body	❑	❑	❑
	Why it is important to seek help quickly for possible signs of cancer	❑	❑	❑
	How to seek help for possible signs of cancer	❑	❑	❑
	Tackling worries or concerns you might have about getting help	❑	❑	❑

3. Your views on getting advice about **cancer screening***, sometime after having an urgent referral appointment with hospital specialists and cancer has not been found.

**Cancer screening means tests that aim to detect cancer early, before a person might have symptoms. There are three NHS cancer screening programmes in England (bowel, cervical and breast screening).*

3a Would you have been willing to receive advice about **cancer screening**, sometime after your urgent referral appointment?

No, definitely not	No, probably not	Not sure	Yes, probably	Yes, definitely
❑	❑	❑	❑	❑

3b Which of the following would you want to receive advice about?	Yes, I would want to receive advice about this	No, I would not want to receive advice about this	Not applicable
Checking you are up-to-date with cancer screening	❑	❑	❑
Help getting a screening kit or screening appointment if needed	❑	❑	❑
Why cancer screening is important	❑	❑	❑
Bowel cancer screening	❑	❑	❑
Breast cancer screening	❑	❑	❑
Cervical cancer screening	❑	❑	❑
Anything else (please add)………..			

3c Receiving advice about **cancer screening**, sometime after my urgent referral appointment...	Strongly disagree	Disagree	Neither agree or disagree	Agree	Strongly agree
...would have made me feel anxious	❑	❑	❑	❑	❑
...would have made me feel judged	❑	❑	❑	❑	❑
...would have made me fearful	❑	❑	❑	❑	❑
...would have made me feel embarrassed	❑	❑	❑	❑	❑
...would have taken too much time	❑	❑	❑	❑	❑
...would have reassured me	❑	❑	❑	❑	❑
...has benefits that outweigh the costs *	❑	❑	❑	❑	❑
...would not have been a priority for me	❑	❑	❑	❑	❑
...would have been stressful for me	❑	❑	❑	❑	❑
...is important	❑	❑	❑	❑	❑
...is a good idea	❑	❑	❑	❑	❑
...would make me more likely to take part in cancer screening	❑	❑	❑	❑	❑
...makes sense to me	❑	❑	❑	❑	❑

* benefits/costs to you or the NHS

3d	When would you have liked to receive advice about **cancer screening**?	*(Tick **Yes** or **No** for each option)*	Yes	No
		With my urgent referral results	❑	❑
		2-4 weeks after receiving my results	❑	❑
		1-3 months after receiving my results	❑	❑
		More than 3 months after receiving my results	❑	❑
		Not at all	❑	❑

3e	How would you have liked to receive advice about **cancer screening**?	*(Tick **Yes** or **No** for each option)*	Yes	No
		Text message with links to videos and/or websites	❑	❑
		Text message chat with a healthcare professional	❑	❑
		In an email	❑	❑
		Leaflet	❑	❑
		Group session with other patients	❑	❑
		In a phone app	❑	❑
		Phone appointment with a healthcare professional	❑	❑
		Video appointment with a healthcare professional	❑	❑
		Face to face appointment with a healthcare professional	❑	❑
		Other (please specify)		

3f	Who would you have liked to give you advice about **cancer screening**?	*(Tick **Yes** or **No** for each option)*	Yes	No
		A doctor from my GP practice	❑	❑
		A nurse or other healthcare professional from my GP practice	❑	❑
		A doctor from the urgent referral clinic	❑	❑
		A nurse or other healthcare professional from the urgent referral clinic	❑	❑
		Somebody else (please specify)		

3g	Have you received any of the following advice about **cancer screening**, since your urgent referral appointment?	Yes	No	Unsure
	Checking you are up-to-date with cancer screening	❑	❑	❑
	Help getting a screening kit or screening appointment if needed	❑	❑	❑
	Why cancer screening is important	❑	❑	❑
	Bowel cancer screening	❑	❑	❑
	Breast cancer screening	❑	❑	❑
	Cervical cancer screening	❑	❑	❑

4. Your views on getting advice about **how to reduce the chances of developing cancer,** sometime after having an urgent referral appointment with hospital specialists and cancer has not been found.

4a Would you have been willing to receive advice about **how to reduce the chances of developing cancer**, sometime after your urgent referral appointment?

No, definitely not	No, probably not	Not sure	Yes, probably	Yes, definitely
❑	❑	❑	❑	❑

4b

Which of the following would you want to receive advice about?	Yes, I would want to receive advice about this	No, I would not want to receive advice about this	Not applicable
Your risk (likelihood) of developing cancer in the future	❑	❑	❑
The types of cancer that you are most at risk of developing	❑	❑	❑
How to stop tobacco use to reduce the chances of developing cancer	❑	❑	❑
How to make changes to your diet, to reduce the chances of developing cancer	❑	❑	❑
How to increase the amount of exercise you do, to reduce the chances of developing cancer	❑	❑	❑
How to reduce the amount of alcohol you drink, to reduce the chances of developing cancer	❑	❑	❑
Anything else (please add)………..			

4c

Receiving advice about **how to reduce the chances of developing cancer**, sometime after my urgent referral appointment...	Strongly disagree	Disagree	Neither agree or disagree	Agree	Strongly agree
...would have made me feel anxious	❑	❑	❑	❑	❑
...would have made me feel judged	❑	❑	❑	❑	❑
...would have made me fearful	❑	❑	❑	❑	❑
...would have made me feel embarrassed	❑	❑	❑	❑	❑
...would have taken too much time	❑	❑	❑	❑	❑
...would have reassured me	❑	❑	❑	❑	❑
...has benefits that outweigh the costs *	❑	❑	❑	❑	❑
...would not have been a priority for me	❑	❑	❑	❑	❑
...would have been stressful for me	❑	❑	❑	❑	❑
...is important	❑	❑	❑	❑	❑
...is a good idea	❑	❑	❑	❑	❑
...would make me more likely to make changes to reduce my chances of developing cancer	❑	❑	❑	❑	❑
...makes sense to me	❑	❑	❑	❑	❑

* benefits/costs to you or the NHS

4d	When would you have liked to receive advice about **how to reduce the chances of developing cancer**?	*(Tick **Yes** or **No** for each option)*	Yes	No
		With my urgent referral results	❑	❑
		2-4 weeks after receiving my results	❑	❑
		1-3 months after receiving my results	❑	❑
		More than 3 months after receiving my results	❑	❑
		Not at all	❑	❑

4e	How would you have liked to receive advice about **how to reduce the chances of developing cancer**?	*(Tick **Yes** or **No** for each option)*	Yes	No
		Text message with links to videos and/or websites	❑	❑
		Text message chat with a healthcare professional	❑	❑
		In an email	❑	❑
		Leaflet	❑	❑
		Group session with other patients	❑	❑
		In a phone app	❑	❑
		Phone appointment with a healthcare professional	❑	❑
		Video appointment with a healthcare professional	❑	❑
		Face to face appointment with a healthcare professional	❑	❑
		Other (please specify)		

4f	Who would you have liked to give you advice about **how to reduce the chances of developing cancer**?	*(Tick **Yes** or **No** for each option)*	Yes	No
		A doctor from my GP practice	❑	❑
		A nurse or other healthcare professional from my GP practice	❑	❑
		A doctor from the urgent referral clinic	❑	❑
		A nurse or other healthcare professional from the urgent referral clinic	❑	❑
		Somebody else (please specify)		

4g	Have you received any of the following advice about **how to reduce the chances of developing cancer**, since your urgent referral appointment?	Yes	No	Unsure
	Your risk (likelihood) of developing cancer in the future	❑	❑	❑
	The types of cancer that you are most at risk of developing	❑	❑	❑
	How to stop tobacco use to reduce the chances of developing cancer	❑	❑	❑
	How to make changes to your diet, to reduce the chances of developing cancer	❑	❑	❑
	How to increase the amount of exercise you do, to reduce the chances of developing cancer	❑	❑	❑
	How to reduce the amount of alcohol you drink, to reduce the chances of developing cancer	❑	❑	❑

Section B: Questions about you, your health behaviour and your health

In this section we would like you to tell us a little bit more about yourself including some information about your current health and health behaviours. This information will help us to understand how views differ between different people.

B1 Over the **past month**, how many portions of fruit or vegetables did you usually eat each day? Please include fruit eaten at meal times or as a snack.

Examples of one portion: 1 apple or banana, or a large slice of melon, or small bowl of grapes, or 2 heaped tablespoons of broccoli or carrots, or 3 tablespoons of sweetcorn or peas

0 portions per day	1 portion per day	2 portions per day	3 portions per day	4 portions per day	5 portions per day	More than 5 portions per day
❑	❑	❑	❑	❑	❑	❑

		No, I have never smoked or used tobacco	No, but I used to smoke or use tobacco	Yes, I smoke or use tobacco (every day or occasionally)
B2	Do you smoke or use tobacco?	❑	❑	❑

		Never	Monthly or less	Two to four times a month	Two to three times a week	Four or more times a week
B3	How often do you have a drink containing alcohol?	❑	❑	❑	❑	❑

		None, I do not drink	1 or 2	3 or 4	5 or 6	7 to 9	10 or more
B4	How many alcoholic drinks do you have on a typical day when you are drinking?	❑	❑	❑	❑	❑	❑

		Never	Less than monthly	Monthly	Weekly	Daily or almost daily
B5	How often do you have six or more alcoholic drinks on one occasion?	❑	❑	❑	❑	❑

B6 In the **past week**, on how many days have you done a total of 30 minutes or more of physical activity, which was enough to raise your breathing rate?

This may include sport, exercise, and brisk walking or cycling for recreation or to get to and from places, but should not include housework or physical activity that is part of your job.

None	1 day	2 days	3 days	4 days	5 days	6 days	7 days
❑	❑	❑	❑	❑	❑	❑	❑

B7	Have you ever been screened for bowel cancer in the last 2 years? *There are two types of tests that have been used in NHS bowel screening: (1) a home testing kit where you provide a sample of your poo, (2) bowel scope screening which involves a thin flexible tube with a camera at the end that looks for and removes any polyps inside your bowel.*			
	Yes, a home testing kit	Yes, bowel scope screening	No	Don't know
	❑	❑	❑	❑

B8	How many times have you had an appointment with a GP in the past year? (your best estimate is fine)	____________________

B9	Have you ever had any of the following conditions? *(Tick all that apply)*				
		❑	Heart disease	❑	Diabetes
		❑	Lung disease or breathing condition (for instance asthma or COPD)	❑	Gastrointestinal condition (problems with your stomach)
		❑	Joint or bone condition (for instance arthritis)	❑	Anxiety or depression

B10	Have you ever been diagnosed with cancer?						
	No	Yes 0-3 months ago	Yes 4-12 months ago	Yes 1-2 years ago	Yes Over 2 years ago	Yes Over 5 years ago	Prefer not to say
	❑	❑	❑	❑	❑	❑	❑

B11	What is your age today?

B12	What is your marital status?				
	Single	Married or civil partner	Separated or divorced	Widowed	Prefer not to say
	❑	❑	❑	❑	❑

B13	What was the highest level of educational qualification you have obtained?		
		❑	No qualifications
		❑	GCSE/ O Level/ CSE
		❑	Vocational qualifications (NVQ1+2)
		❑	A Level or equivalent (NVQ 3)
		❑	Bachelor degree or equivalent (NVQ 4)
		❑	Masters/ PhD or equivalent
		❑	Other

B14	What is your postcode?

B15	What is your ethnic group?	
White: English/ Welsh/ Scottish/ Northern Irish/ British	❑	
White: Irish	❑	
White: Gypsy/ Irish Traveller	❑	
Any other White background	❑	Please specify:
Multiple ethnic group: White and Black Caribbean	❑	
Multiple ethnic group: White and Black African	❑	
Multiple ethnic group: White and Asian	❑	
Any other Mixed/Multiple ethnic group	❑	Please specify:
Asian/ Asian British: Indian	❑	
Asian/ Asian British: Pakistani	❑	
Asian/ Asian British: Bangladeshi	❑	
Asian/ Asian British: Chinese	❑	
Any other Asian background	❑	Please specify:
African/ Caribbean/ Black British: African	❑	
African/ Caribbean/ Black British: Caribbean	❑	
Any other African/ Caribbean/ Black British	❑	Please specify:
Other ethnic group: Arab	❑	
Any other ethic group	❑	Please specify:

Thank you for participating in this study.

Please return this questionnaire in the envelope provided.

If you have any questions please contact: ruth.e.evans@kcl.ac.uk

If you have any new or continuing symptoms since your urgent referral appointment we encourage you to speak to your doctor.
